# Novel NAC-loaded poly(lactide-co-glycolide acid) nanoparticles for cataract treatment: preparation, characterization, evaluation of structure, cytotoxicity, and molecular docking studies

**DOI:** 10.7717/peerj.4270

**Published:** 2018-01-30

**Authors:** Yasemin Budama-Kilinc, Rabia Cakir-Koc, Serda Kecel-Gunduz, Yagmur Kokcu, Bilge Bicak, Hande Mutlu, Aysen E. Ozel

**Affiliations:** 1 Department of Bioengineering, Yildiz Technical University, Istanbul, Turkey; 2 Physics Department, Istanbul University, Istanbul, Turkey; 3 Graduate School of Engineering and Sciences, Istanbul University, Istanbul, Turkey

**Keywords:** NAC, Molecular dynamic, Zeta-sizer, GROMACS, TEM, FTIR, Molecular docking, Nanoparticle, PLGA

## Abstract

**Background:**

*N*-acetylcarnosine (NAC), a dipeptide with powerful antioxidant properties that is extensively used as a pharmaceutical prodrug for the treatment of cataract and acute gastric disease, was investigated by molecular dynamics with the GROMACS program in order to understand the solvent effect on peptide conformation of the peptide molecule used as a component of a drug and which presents substantial information on where drug molecules bind and how they exert their effects. Besides, molecular docking simulation was performed by using the AutoDock Vina program which identify the kind of interaction between the drug and proteins. A delivery system based on poly(lactic-co-glycolic acid) (PLGA) nanoparticles (NPs) loaded with NAC (NAC-PLGA-NPs) for the treatment of cataract was prepared for the first time in this study in order to enhance drug bioavailability and biocompatibility. The objective of this work was to prepare and evaluate the structural formulation, characterization, and cytotoxicity studies of NAC-loaded NPs based on PLGA for cataract treatment.

**Methods:**

PLGA and NAC-loaded PLGA NPs were prepared using the double emulsion (w/o/w) method, and characterizations of the NPs were carried out with UV–Vis spectrometer to determine drug concentration, the Zeta-sizer system to analyze size and zeta potential, FTIR spectrometer to determine the incorporation of drug and PLGA, and TEM analysis for morphological evaluation.

**Results:**

NAC-loaded PLGA NPs were successfully obtained according to UV–Vis and FTIR spectroscopy, Zeta-sizer system. And it was clearly observed from the TEM analysis that the peptide-loaded NPs had spherical and non-aggregated morphology. Also, the NPs had low toxicity at lower concentrations, and toxicity was augmented by increasing the concentration of the drug.

**Discussion:**

The NAC molecule, which has been investigated as a drug molecule due to its antioxidant and oxidative stress-reducing properties, especially in cataract treatment, was encapsulated with a PLGA polymer in order to increase drug bioavailability. This study may contribute to the design of drugs for cataract treatment with better reactivity and stability.

## Introduction

The number of visually impaired people in the world is estimated to be 253 million according to the World Health Organization (WHO). Cataracts are the main cause of this visual impairment in cases of moderate and low severity (WHO, 2014). According to a WHO assessment in 2010, cataracts were responsible for 51% of the cases of blindness in the world, representing approximately 20 million cases. Cataracts inhibit the passage of light by clouding the eye lens. Although most of these cases are due to age, it is possible for children to be born with this condition, and it can occur after an injury, inflammation, or disease (WHO, 2010).

Cataracts can be treated with surgery, but Babizhayev and his team have shown that an eye solution containing 1% *N*-acetylcarnosine (NAC) can be used as an ophthalmic prodrug in the treatment of senile cataracts (Babizhayev et al., 2001).

*N*-acetylcarnosine contains beta alanine and histidine amino acids in its structure and is bearing a natural imidazole ring (Babizhayev, 2006). In aqueous media, NAC, a relatively soluble molecule in lipids, easily passes through the lipid membranes and corneal tissue which consequently provides its easy access to the intraocular environment (Babizhayev, 1996).

In addition to being used as an ophthalmic prodrug in senile cataracts, the antioxidant effect has also led to studies on human skin. Nino, Iovine & Santoianni (2011) studied the antioxidant and photoprotective effects of carnosine and NAC on human skin before and after UVB irradiation and found that the NAC solution caused a 7.3% decrease in erythema. In a study on LPS-induced microglial cells, it was determined that the NAC molecule could regulate microglial inflammation and oxidative stress (Fleisher-Berkovich et al., 2009).

Drug-release systems provide a great advantage for drug molecules with protein structures that have a short half-life. Poly(lactic-co-glycolic acid) (PLGA), which is a biodegradable polymer most commonly used in drug-release systems, is the most preferred polymer in synthesis of nano drug delivery systems because of its biocompatibility and biodegradability. It is known that PLGA-based NPs increase the bioavailability of drugs that are not absorbed efficiently (Brannon-Peppas, 1995; Jalil & Nixon, 1990; Yeh, Davis & Coombes, 1996).

Although there have been studies regarding the NAC molecule, no studies have been based on the encapsulation of the NAC molecule. Hence, the main purpose of this study is to prepare and characterize the NAC-loaded PLGA NPs (NAC-PLGA-NPs) for treating ophthalmic diseases such as cataracts.

## Materials and Methods

### Materials

*N*-acetylcarnosine was commercially purchased from Toronto Research Chemicals (Toronto, Ontario Canada) with >98% purity. The molecular weight of the NAC was 268.27 g mol^−1^. PLGA (lactide:glicolide = 50:50, *M*_w_ ∼ 38–54 kDa) and poly(vinyl alcohol) (PVA) were purchased from Sigma-Aldrich (St. Louis, MO, USA). Dichloromethane (DCM) was also purchased from Merck Millipore (>99.5%) (Darmstadt, Germany). All the chemicals and solvents were of analytical grade. Ultrapure water was obtained using the Millipore MilliQ Gradient System. Molecular dynamics (MD) simulations were performed using GROMACS code (Van Der Spoel et al., 2005) for 5 ns.

### Nanoparticle preparation method

Blank PLGA and NAC-PLGA-NPs were prepared using the double emulsion (w/o/w) method. PLGA was dissolved in DCM. NAC was dissolved in ultrapure water and then added to the polymer solution. The mixture was homogenized using probe sonication (Bandelin, Berlin, Germany) at 50 W for 5 min in an ice bath. Then, 2.5% PVA was dissolved in distilled water and added to the mixture, before sonication step. The double emission formed was gently stirred at room temperature overnight to evaporate the organic phase. The NAC-PLGA-NPs were purified by applying three cycles of the centrifugation step (10,000 rpm for 20 m at +4 °C in Hettich (Tuttlingen, Germany) centrifuge) and then washed for three cycles with ultrapure water to remove the excess amount of PVA and non-trapped drug molecules. Finally, the NPs were filtered through a 0.45 μm regenerated cellulose membrane. Blank PLGA-NPs were also prepared by the same method without adding NAC at any step of the preparation.

### Determination of encapsulation and loading efficiency

The standard calibration curve of NAC was obtained by a UV–Vis spectrometer at 220 nm to calculate the encapsulation efficiency. By separating the NPs from the aqueous NP suspension after the centrifugation step, the encapsulation efficiency of NAC-PLGA-NPs was determined by calculating the concentration of the free NAC quantity in the supernatant from the NAC standard calibration curve. PLGA NPs was used as blank.

$${\rm{Encapsulation\;efficiency}} = {{{\rm{Total\;drug\;amount}}-{\rm{Free\;drug\;amount}}} \over {{\rm{Total\;drug\;amount}}}} \times 100$$

The loading efficiency of NAC was determined by measuring the weight of the freeze-dried NPs using the following equation:

$${\rm{Loading\;efficiency}} = {{{\rm{Encapsulated\;drug\;amount}}} \over {{\rm{Nanoparticle\;weight}}}} \times 100$$

### Dynamic light scattering and zeta potential analysis

To determine the size, polydispersity index (PdI), size distribution, and zeta potential of NPs, the solutions were transferred into transparent cuvettes. Blank NPs and NAC-loaded NPs were measured using the Zetasizer Nano ZS (Malvern Instruments, Malvern, UK) instrument, equipped with a 4.0 mV He–Ne laser (633 nm) at a temperature of 25 °C, which was capable of both particle size analysis (using dynamic light scattering (DLS) as the basic principle of operation) and zeta-potential measurement (using Doppler electrophoresis as the basic principle of operation). Before every measurement, each sample was filtered with 0.2 μm regenerated cellulose membrane (Sartorius, Göttingen, Germany) filters to remove the aggregates from the solutions.

### Fourier transform infrared spectroscopy

The infrared spectrum gives detailed information about functional groups and where they are presented in a particular drug formulation. IR spectra of NAC, PLGA, and NAC-PLGA-NPs were recorded by using an IR Prestige 21 Fourier transform infrared (FT-IR) spectrophotometer with total reflectance (ATR) mode (Shimadzu, Kyoto, Japan) for characteristic analyses of the functional groups. The peaks in ATR spectrum were interpreted and compared to literature values.

### MD simulations

The MD simulations were carried out using the GROMACS v5.2 software package (Van Der Spoel et al., 2005) for 5 ns. The initial conformation of NAC was obtained from the result of optimized geometry, which was calculated at DFT/B3LYP level of theory with the 6-31++G (d,p) basis set by using the Gaussian program (Gaussian 09: Frisch et al., 2009). The parameters of the NAC dipeptide were created by using the ATB database online server (https://atb.uq.edu.au/) that used the GROMOS 54A7 force field. The peptide was confined to a cubic box of simple point charge (SPC) water molecules (Smith & van Gunsteren, 1993) with 2,317 generated water molecules, and four Na^+^ and four Cl^−^ ions were added to neutralize the whole system. Simulation was performed with a time step of 2 fs and periodic boundary conditions in all three directions. The system was subjected to energy minimization by using the steepest descent method. Then, NVT (constant number of atoms, volume, and temperature) and NPT (constant number of atoms, pressure, and temperature) ensembles were used to yield equilibration. The whole system was stabilized to 310 K using a Berendsen thermostat (Berendsen et al., 1984) at a pressure of 1 bar and compressibility of 4.5 × 10^−5^ bar^−1^ using an isotropic Parrinello–Rahman barostat (Parrinello & Rahman, 1981). In addition, the NVT and NPT simulations were performed at 100 and 500 ps to bring the system to the target temperature and determine the correct density. On the other hand, in order to examine the time-dependent trajectories, Newton’s second equation of motion was integrated with the leapfrog algorithm with a time step of 2 fs. This procedure was followed by MD production run to generate trajectory data for analysis. For the simulation, the Particle Mesh Ewald method was used to calculate the long-range electrostatic interaction with a 0.12 nm grid width and a fourth-order cubic interpolation (Darden, York & Pedersen, 1993). The linear constraint solver (LINCS) algorithm (Hess et al., 1997) was used to constrain all the bonds. The short-range electrostatic and van der Waals interactions were described by using the Verlet cut-off scheme (Verlet, 1967) with a 0.8 nm cut-off radius, and they were updated for each time step. Initial velocities were assigned randomly from the Maxwell distribution at 310 K. At the same time, coordinates and energies were recorded at every step for the trajectory analysis.

### HOMO–LUMO and UV–Vis analysis

The UV–Vis absorption spectra of the NAC solutions (in methanol and distilled water) were recorded using the Shimadzu UV-1280 UV–Vis recording spectrometer in the spectral region of 200–700 nm. The experimentally obtained excitation energies (*E*), absorbance wavelengths (λ), and absorbance values of NAC molecules are tabulated in Table 1A. Besides, the calculated absorption wavelengths λ (nm), excitation energies *E* (eV), and oscillator strengths (*f*) of NAC molecules along with transition levels and assignments in water and methanol solvents were obtained in the framework of TD-DFT calculations with the B3LYP/6-311++G (d,p) method by using the Gaussian program (Frisch et al., 2009), and they are tabulated in Table 1B.

**Table 1:** Experimentally and calculated obtained absorption wavelength and absorbance values of NAC molecule.

(A) Experimentally obtained absorption wavelength λ (nm), excitation energies *E* (eV), and absorbance values of NAC molecule in several solutions			
	*E* (eV)	λ (nm)	Abs.
Methanol	5.510	225.0	2.727
dH_2_O	5.700	217.5	3.215
	5.834	212.5	3.089
	5.989	207.0	3.167

(B) Calculated absorption wavelengths λ (nm), excitation energies *E* (eV), and oscillator strengths (*f*) of NAC along with transition levels and assignments in various solvents						
TD-B3LYP/6-311++G (d,p)						
	*E* (eV)	λ (nm)	*f*	*Major contribution*		
Methanol	4.8971	253.18	0.0199	H → L	(97%)	σ → σ^*^
	5.3708	230.85	0.0073	H → L + 1	(66%)	σ → σ^*^
				H → L + 2	(31%)	
	5.3934	229.88	0.0378	H → L + 1	(30%)	σ → σ^*^
				H → L + 2	(57%)	
dH_2_O	4.9081	252.61	0.0201	H → L	(97%)	σ → σ^*^
	5.3871	230.15	0.0066	H → L + 1	(52%)	σ → σ^*^
				H → L + 2	(43%)	
	5.4085	229.24	0.0367	H → L + 1	(42%)	σ → σ^*^
				H → L + 2	(40%)	

### In vitro cell culture

The L929 cell line was used for in vitro toxicity experiments. L929 (mouse fibroblast cells) are commercially available from ATTC (https://www.lgcstandards-atcc.org/products/all/CCL-1.aspx?geo_country=tr), and ethical approval is not necessary for the standard cell lines. We did not use any animals to obtain the cell line. L929 cells were cultured in DMEM-F12 medium (supplemented with 10% fetal bovine serum and penicillium–streptomycin: 0.5% from 10,000 units ml^−1^ Penicillin–10 mg ml^−1^ Streptomycin) and incubated at 37 °C in 5% CO_2_. Confluent culture was trypsinizated to detach cells from the surface and centrifuged at 5,000 rpm for 5 min. The supernatant was discarded, and the cell number in the pellet was counted with a hemocytometer. Subsequently, a 10^5^ cells ml^−1^ concentration of L929 cells was prepared for toxicity experiments.

### XTT assay for toxicity

The L929 cells with the concentration of 10^5^ in 1 ml of medium were seeded in 96-well flat-bottom microplates and incubated at 37 °C for 24 h for cell attachment. Different concentrations of NAC and NAC-PLGA-NPs in distilled water were added to the wells (*n* = 5) with peptide concentrations of 2, 8, 14, 20, 30, 40, 60, 80, and 100 μg ml^−1^, and distilled water was used as a negative control. Briefly, same volume of culture media was aspirated and then samples were added. Cells were incubated for 24 h, and the medium was replaced with 100 μl fresh medium containing 2,3-bis-(2-methoxy-4-nitro-5-sulfophenyl)-2*H*-tetrazolium-5-carboxanilide (XTT) with a concentration of 0.5 mg ml^−1^ (with 7.5 μg ml^−1^ phenazine methosulfate). The plates were incubated for 4 h at 37 °C, and optical density was measured at 450 nm (Thermo Labsystems Multiskan Ascent 354 Microplate Photometer). The percentage cell viability was calculated according to the following formula:

$${\rm{\% }}\,{\rm{Cell\;viability}} = {{{\rm{Optical\;density\;of\;sample}}} \over {{\rm{Optical\;density\;of\;control}}}} \times 100$$

### Transmission electron microscopy analysis

Nanoparticle morphology was analyzed using a transmission electron microscope (JEOL TEM 1400 Plus) at 80 kV. The NP samples were placed directly into an ultrasound bath at room temperature for 1–2 min, and then two or three drops of the solution were deposited on a Formvar-coated carbon-supported copper grid and transmission electron microscopy (TEM) images were observed.

## Results and Discussion

### MD results

**Figure 1:**
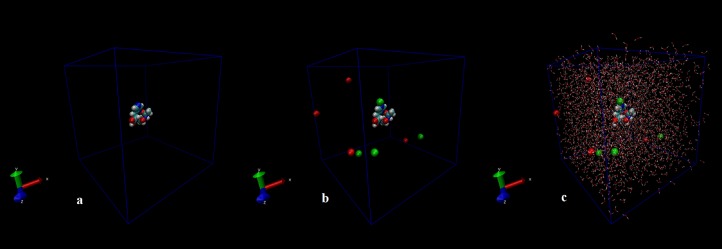
Initial conformation of *N*-acetylcarnosine molecule (A), in a cubic box solvated with four Na^+^ and four CL^−^ ions (B), in a cubic box solvated with 2,317 water molecules and eight ions (C).

**Figure 2:**
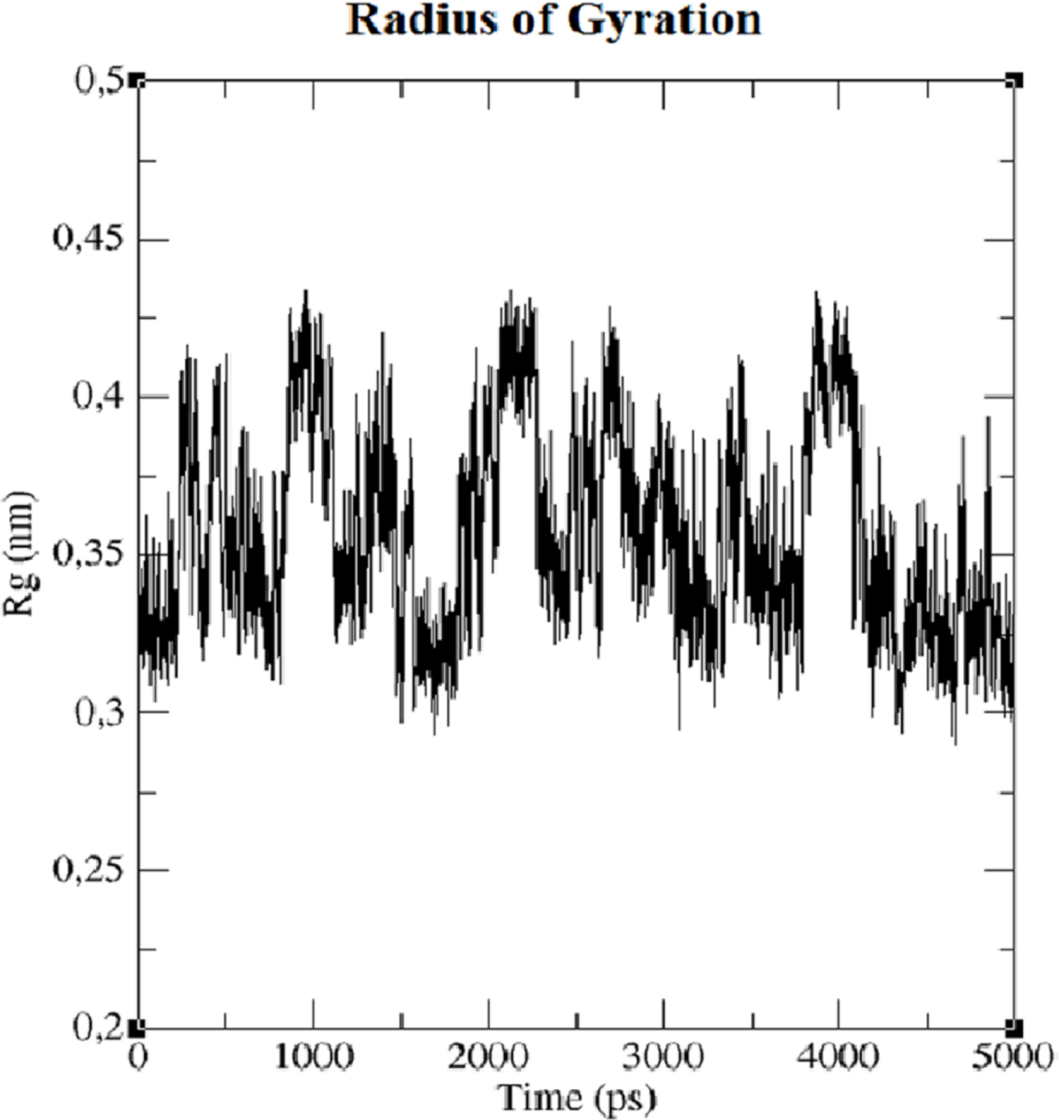
Radius of gyration (Rg) of system.

**Figure 3:**
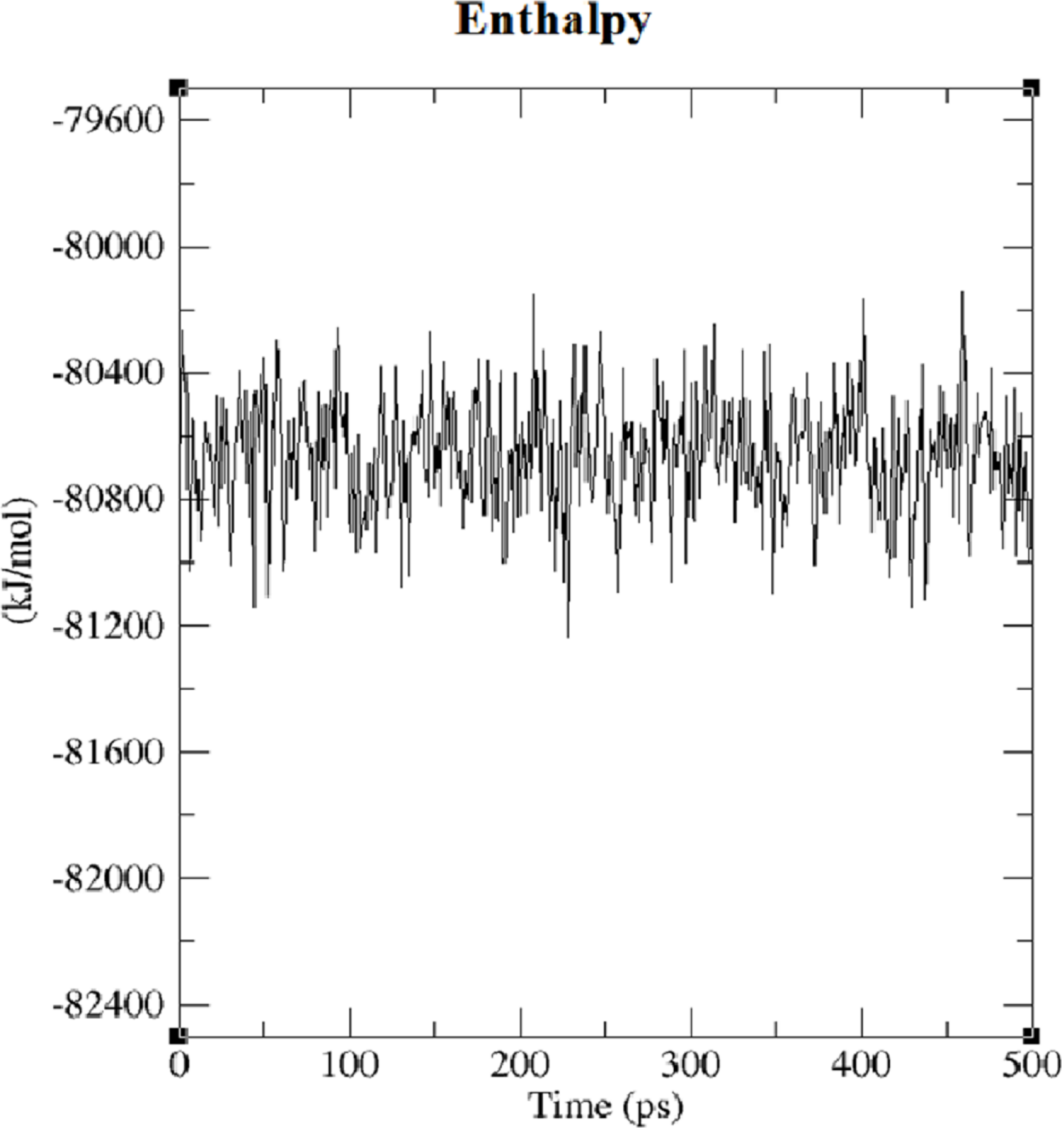
Enthalpy of system solvated with 2,317 water molecules and eight ions.

The first structure of the dipeptide NAC was generated using an ideal geometry into a cubic box with 2,317 water molecules of the SPC water model. Counter ions Na^+^ and Cl^−^ were added to neutralize the system (see Fig. 1). The solvated system was subjected to energy minimization with the steepest-descent algorithm for 50,000 steps to provide the appropriate geometry. The potential energy in the system converged to a large negative number at −1.08176 × 10^5^ kJ mol^−1^ for the solvated system with 2,317 water molecules and eight counter ions. Figure S1 shows that energy minimization occurred properly. After the structure of minimized energy, temperature (310 K) and pressure (1 bar) stabilized with the NVT and NPT ensembles, respectively. NVT was carried out for 50,000 steps with a 2 fs time step, and a Berendsen thermostat was used for coupling the temperature. The NVT results show that the system was well equilibrated around the target temperature at 310 K. NPT calculation was performed at total 500 ps by 250,000 steps with a 2 fs time step. The systems were subjected to a Parrinello–Rahman barostat to couple pressure isotropically (the same in all directions) to a value of 1.0 bar. The average density of the NPT simulation was obtained as 975 kg m^−3^ (see Fig. S2). MD simulation was performed to equilibrate the system for 5 ns (5,000 ps) with a 2 fs timestep (2,500,000 steps). After the 5 ns MD simulation, root mean square deviations (RMSDs) were calculated for NAC molecules, as shown in Fig. S3. RMSD is a measure that shows how much the protein structure changes over the course of the simulation. Changes between 1 and 3 Angstroms are perfectly acceptable (and expected) for small and globular proteins. As shown in Fig. S3, during the simulations, the molecular structure was unfolded and refolded several times. The radius of gyration (Rg) was also calculated to endorse the RMSD results in Fig. 2. Similar fluctuations may be seen in the range of 1,500–2,000 ps in the Rg and RMSD figures. Rg is stable when a protein is stably folded and maintains a relatively stable value of Rg, but if a protein is unfolded, the Rg value will change over time. The enthalpy of the system as a function of MD equilibration is also shown in Fig. 3.

### HOMO–LUMO and UV–Vis analysis results

**Figure 4:**
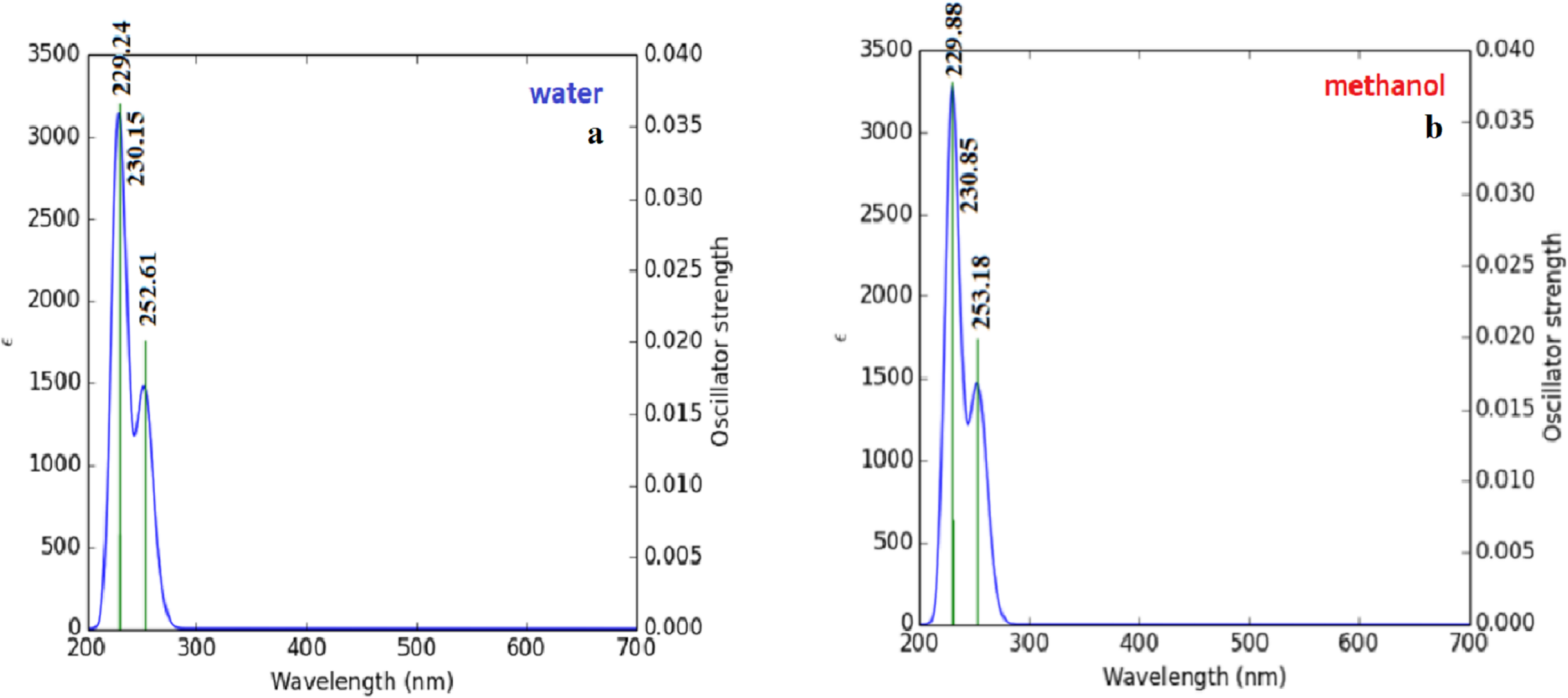
Calculated UV–Vis absorption spectra of NAC peptide (A) in water, (B) in methanol.

**Figure 5:**
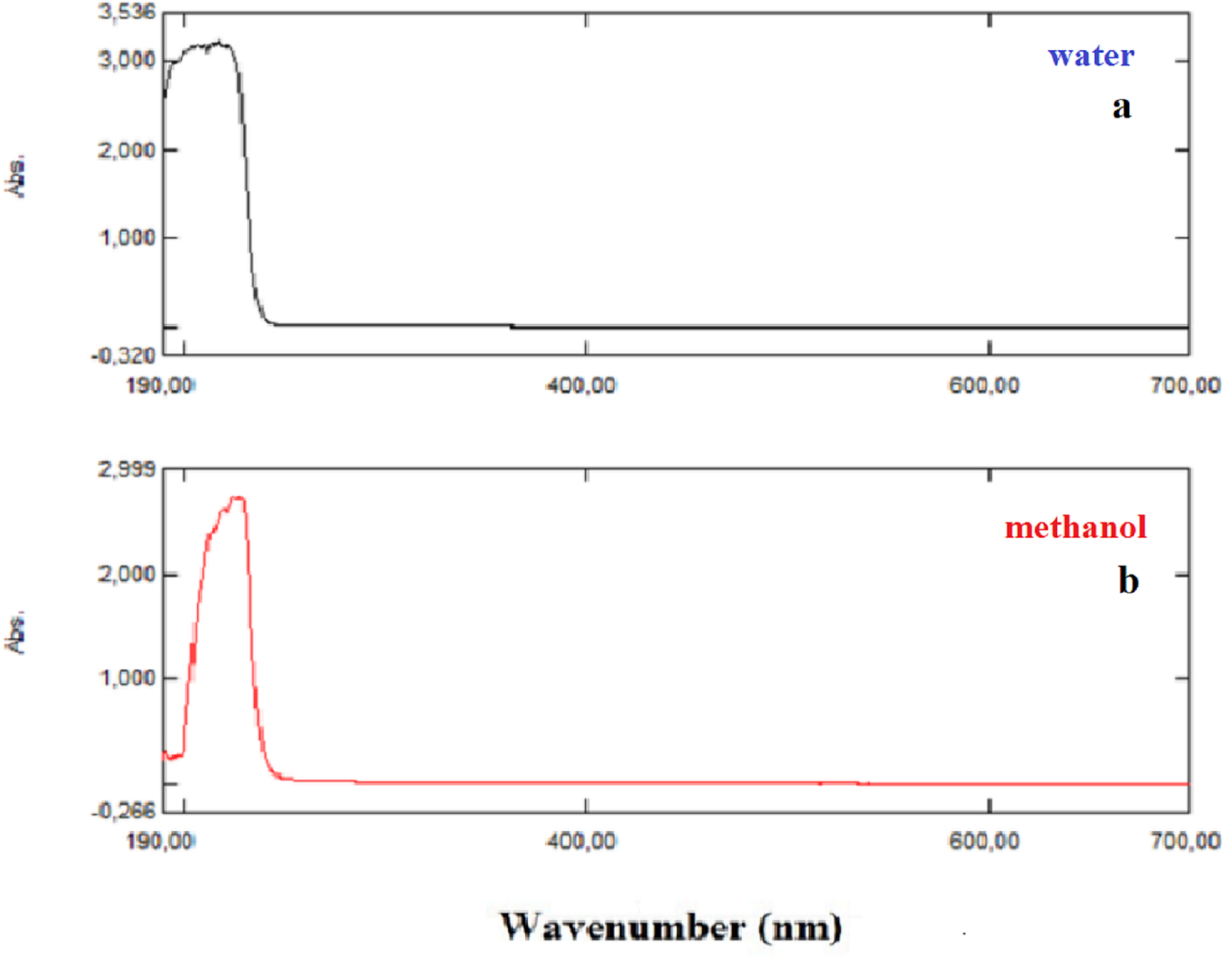
Experimentally recorded UV–Vis absorption spectra of NAC peptide: (A) water, (B) methanol.

**Figure 6:**
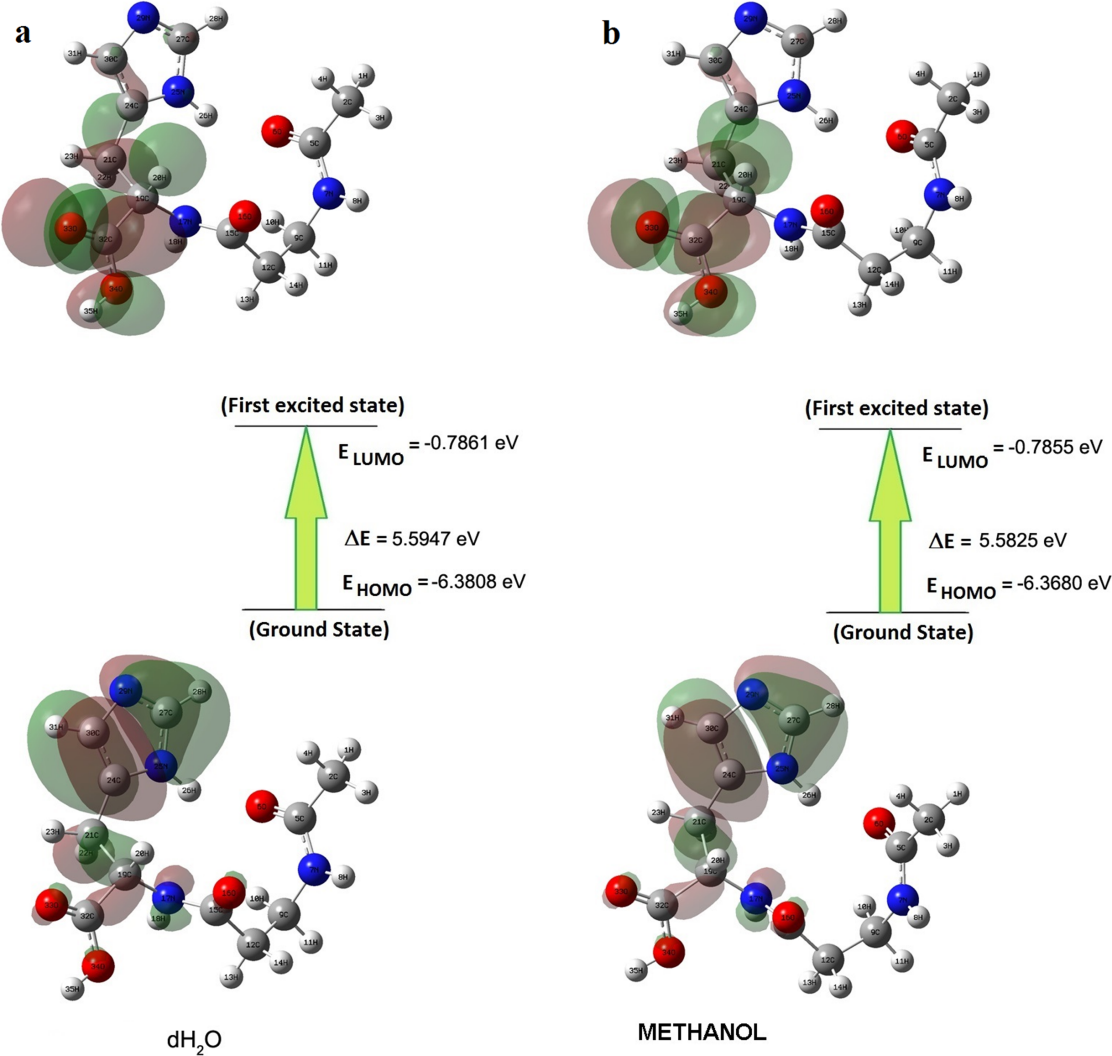
Patterns of principle frontier molecular orbitals of NAC obtained with TD-DFT/B3LYP/6-311++G (d,p) (A) in water, (B) in methanol.

Briquet and Vercauteren noticed that TD-DFT λ_max_ calculations by the 6-311++G (d,p) basis set are appropriate with experimental results (Briquet et al., 2007). The calculated values obtained with B3LYP/6-311++G (d,p) were 253.18, 230.85, and 229.88 nm (in methanol) and 252.61, 230.15, and 229.24 nm (in water), as shown in Fig. 4. The experimentally determined maximum absorption values were 217.5, 212.5, and 207.0 nm (in water) and 225 nm (in methanol), as shown in Fig. 5. According to the calculated UV spectra by using TD-DFT, the greatest absorption wavelength corresponded to the electronic transition from the highest occupied molecular orbital (HOMO) to the lowest unoccupied molecular orbital (LUMO) (Karabacak et al., 2012). The molecular orbital energies (eV) and energy differences of NAC molecules are presented in Table 2. The ionization potential, electron affinity, electronegativity, chemical hardness, chemical softness, and HOMO–LUMO gaps for NAC molecules were calculated, and they are listed in Table 3. The chemical stability and reactivity of a molecule can be evaluated with its hardness and softness calculations. The value of hardness (2.7973 eV) and the larger energy gap (5.59 eV) for the water solution indicated that the molecules were more stable in aqueous media than in methanol. The calculations of the molecular orbital geometry show that the visible absorption maxima of this molecule were 5.51 and 5.70 eV for methanol and water solution, respectively, as shown in Table 1A. These values correspond to the electron transition from HOMO to LUMO values with 5.58 and 5.59 eV for the methanol and water solution, respectively, as shown in Fig. 6. HOMO is formed on the histidine, peptide group and on C_19_–C_32_ atoms in the carboxyl group; LUMO also occurred on the carboxyl group and C_19_–C_21_–C_24_ atoms in the side chain of the histidine part. The HOMO–LUMO transition showed that charge transfer occurred from the imidazole ring in the histidine part to the carboxyl groups and side chain of the histidine part.

**Table 2:** Calculated molecular orbital energies (eV) and energy differences of NAC molecule.

TD-B3LYP/6-311++G (d,p)							
	*E*_LUMO+2_	*E*_LUMO+1_	*E*_LUMO_	*E*_HOMO_	Δ*E*_HOMO–LUMO_	Δ*E*_(HOMO)–(LUMO+1)_	Δ*E*_(HOMO)–(LUMO+2)_
Methanol	−0.3564	−0.4644	−0.7855	−6.3680	5.5825	5.9036	6.0116
dH_2_O	−0.3523	−0.4547	−0.7861	−6.3808	5.5947	5.9261	6.0285

**Table 3:** The calculated values of ionization potential, electron affinity, and HOMO–LUMO gaps for NAC.

TD-DFT/6311++G(d,p)			
Methanol		Energy (a.u.)	Energy (e.V.)
HOMO energy	*E*_HOMO_	−0.23402	−6.3680
LUMO energy	*E*_LUMO_	−0.02887	−0.7855
Ionization potential	*I* = −*E*_HOMO_	0.23402	6.3680
Electron affinity	*A* = −*E*_LUMO_	0.02887	0.7855
Elektronegativity	χ = (*I* + *A*)/2	0.13144	3.5767
Chemical potential	μ = −(*I* + *A*)/2	−0.13144	−3.5767
Chemical hardness	η = (*I* − *A*)/2	0.10257	2.7912
Δ*Ε* (gap)	*E*_LUMO_−*E*_HOMO_	0.20515	5.5825

**dH_2_O**			
HOMO energy	*E*_HOMO_	−0.23449	−6.3808
LUMO energy	*E*_LUMO_	−0.02889	−0.7861
Ionization potential	*I* = −*E*_HOMO_	0.23449	6.3808
Electron affinity	*A* = −*E*_LUMO_	0.02889	0.7861
Elektronegativity	χ = (*I* + *A*)/2	0.13169	3.5834
Chemical potential	μ = −(*I* + *A*)/2	−0.13169	−3.5834
Chemical hardness	η = (*I* − *A*)/2	0.10280	2.7973
Δ*Ε* (gap)	*E*_LUMO_−*E*_HOMO_	0.20560	5.5947

### MEP analysis results

**Figure 7:**
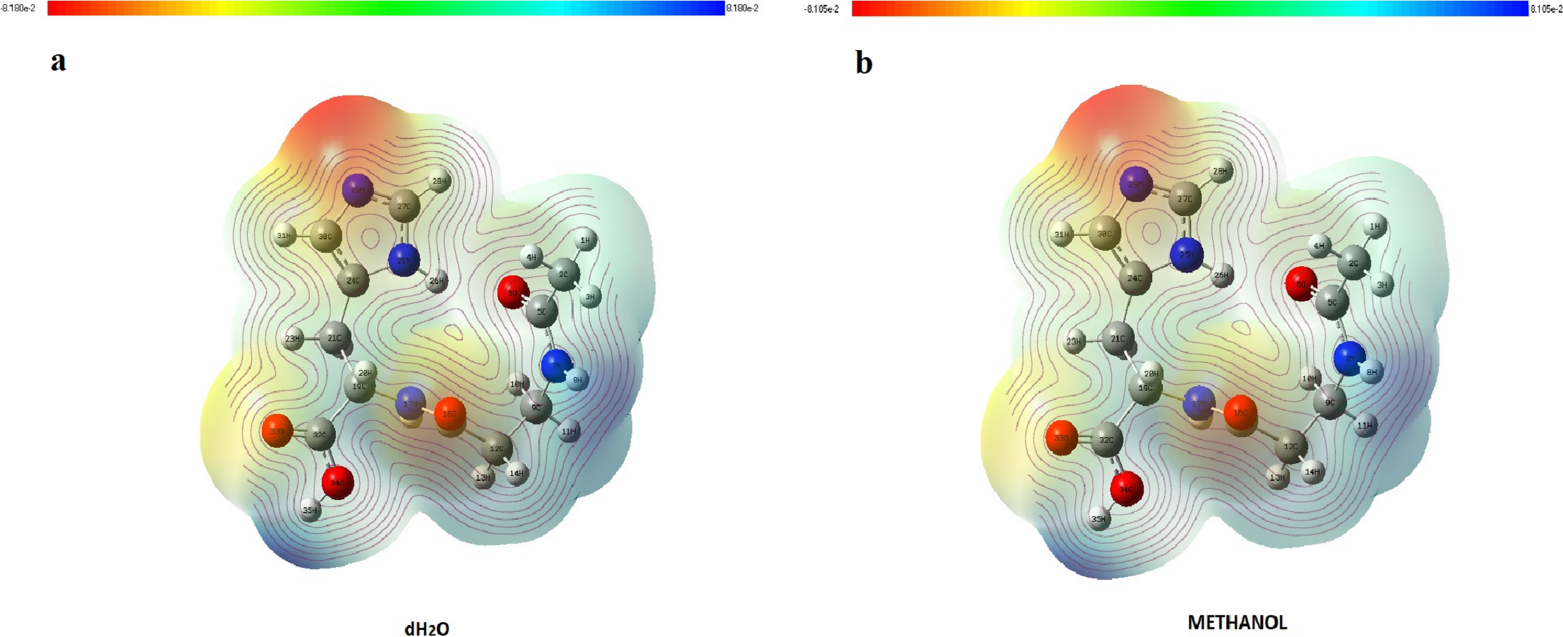
MEP maps of NAC obtained with (A) water solution and (B) methanol solution.

It is very important to determine the location of charge distributions, such as reactivity sites for electrophilic and nucleophilic attacks for NAC molecules. The *V*(*r*) values of MEP maps were concerned with electrophilic reactivity, which had relative abundance, shown as the negative regions of maps, while the nucleophilic reactivity (relative absence of abundance of electrons) are shown as the positive regions. Different values of the electrostatic potential at the surface are represented by different colors. The lowest value of electrostatic potential energy is defined with red, while the highest electrostatic potential energy value is defined with blue, as seen in Fig. 7. MEP analysis results of NAC molecules for methanol solution were colored, with the regions with negative potential (−0.08105 a.u.) over the electronegative atoms (nitrogen atom in histidine imidazol functional group) defined by red, and the regions with positive potential (0.08105 a.u.) over hydrogen atoms (hydrogen atom in carboxyl group and in peptide bond) defined by blue. The maximum negative region, which has a value of −0.0791187 a.u. localized on the nitrogen atom in the histidine imidazol functional group (N_29_), is shown by intense red. Other negative regions were oxygen atoms in the peptide group (O_16_) and in the carboxyl group (O_33_), and their values are defined as −0.056387 and −0.0492807 a.u., respectively. The maximum positive regions of the peptide were defined on hydrogen atoms in the peptide bond (H_18_) with a value of +0.0786242 a.u. Other positive regions were defined as hydrogen atoms again and had values of +0.07611517 a.u. (H_35_ in carboxyl group) and +0.073427 a.u. (H_8_ in peptide group between acetyl group and beta alanine). Similarly, in the methanol results, the same atomic regions were found for negative and positive potentials in the water solution. For the water solution, the deepest negative and positive potentials obtained were −0.08180 and 0.08180 a.u., respectively, as seen in Fig. 7. The negative potentials were localized on N_29_, with a value of −0.0795793 a.u., while −0.0567596 and −0.0497109 a.u. values defined O_16_ (in peptide group) and O_33_ (in carboxyl group), whose regions are the same as in methanol. As expected, positive potentials were localized on hydrogen atoms, which are more electropositive. H_18_ (in peptide group), H_35_ (in carboxyl group), and H_8_ (in peptide bond between acetyl group and beta alanine) had values of +0.081481, +0.0764613, and +0.0737115 a.u., respectively.

### Molecular docking results

**Figure 8:**
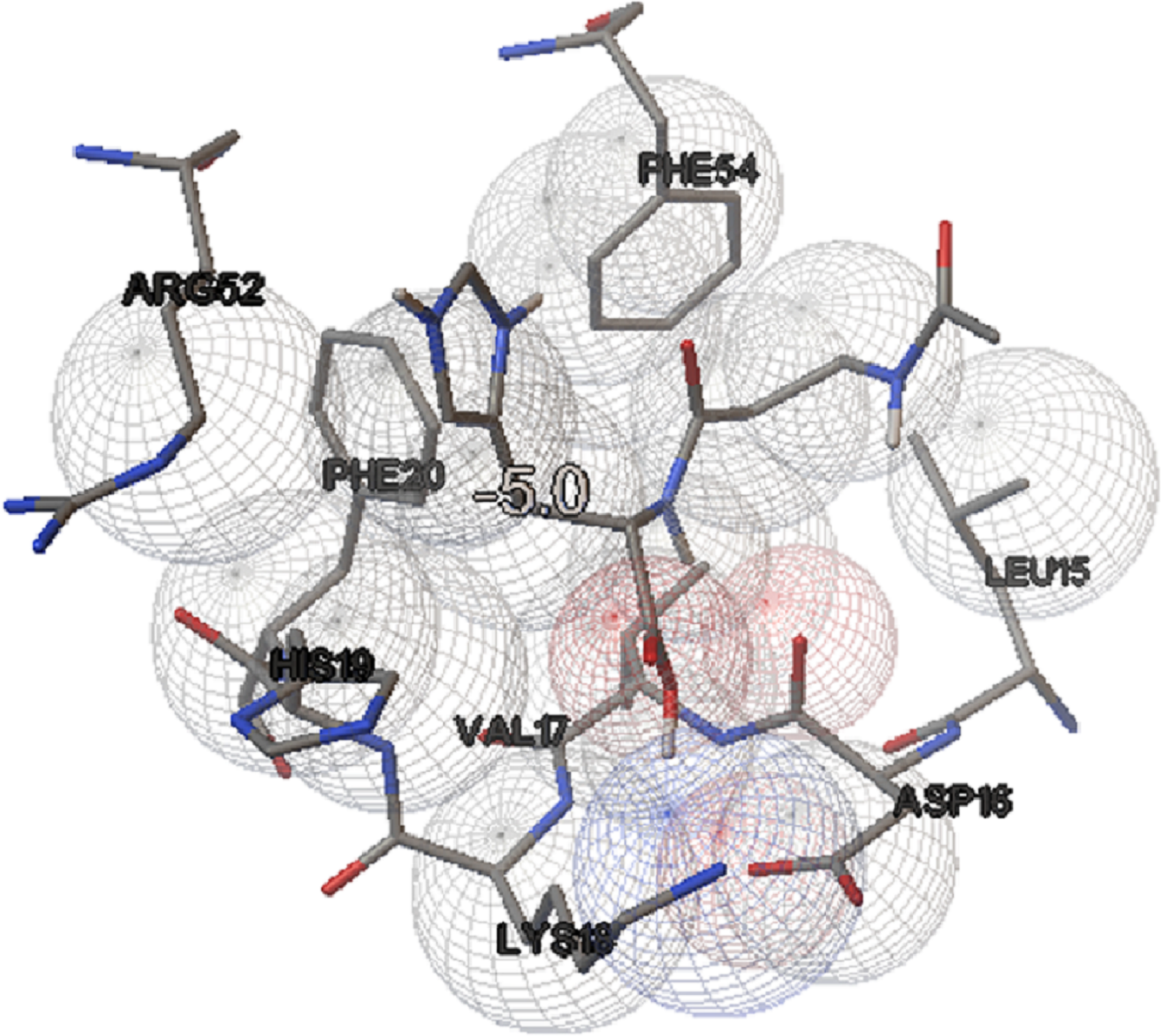
Schematic for docked conformation of active site of title compound.

**Figure 9:**
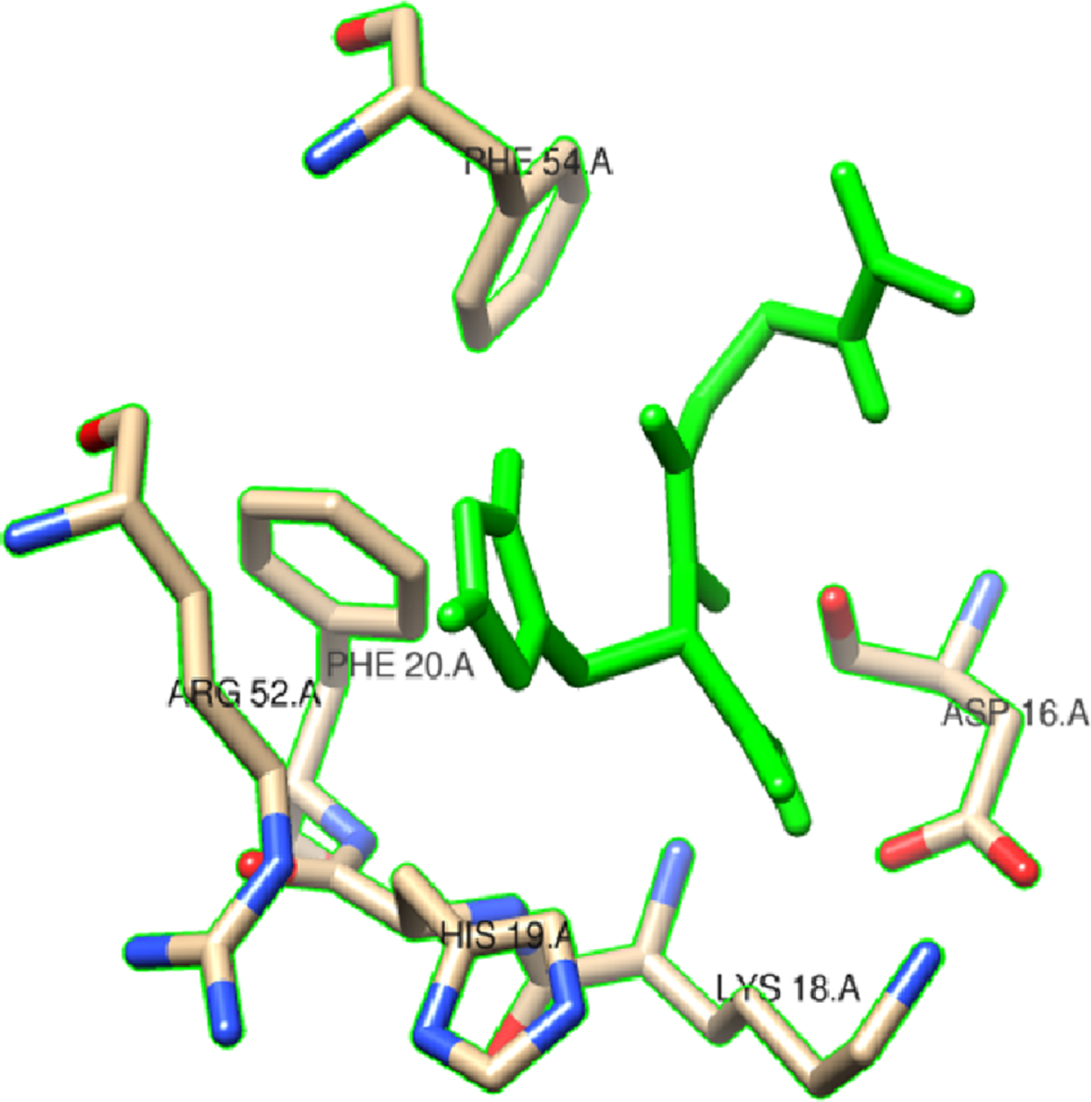
Schematic for docked conformation of ASP-16, VAL-17, and LYS-18 in active site within protein.

**Figure 10:**
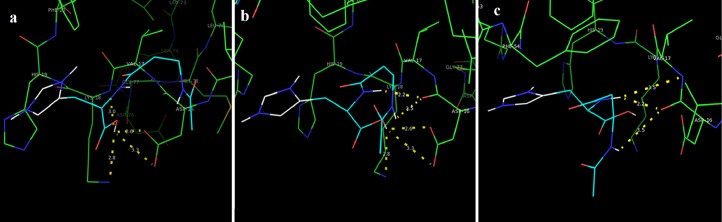
Schematic hydrogen bonding (yellow) for docked conformation of active site of title compound with (A) ASP-16, (B) VAL-17, and (C) LYS-18.

In this study, we examined the AutoDock binding affinities of the NAC molecule onto Bovine alpha A crystalline (Cheng et al., 2009). Alpha A is a major protein ingredient of the mammalian eye lens. As it is a member of the small heat-shock protein family, it possesses a chaperone-like function. The mutations in alpha A can cause cataracts and myopathy (Horwitz, 2003). The crystal structure of Bovine alpha A crystallin, which is structurally expressed in many cells and known to be involved in many diseases (Horwitz, 2009), was chosen as the receptor for the docking simulations. The crystal structure of Bovine alpha A crystallin (PDB code: 3L1F) (Laganowsky et al., 2010) is provided via the Protein Data Bank (http://www.rcsb.org/pdb). For better results, we were able to build our protein homology model by the SWISS-MODEL server (Schwede et al., 2003). The active site of the protein was defined to include residues of the active site within the grid size of 40 × 40 × 40 Å. The protein was primarily prepared by removing the (4*S*)-2-methyl-2,4-pentanediol and water. For the molecular docking calculations, AutoDock Vina software (Trott & Olson, 2010) was chosen to determine an efficient tool for finding the binding poses and binding orientations of the ligands. PyMOL and the Chimera molecular graphics system were utilized for analyzing receptor–ligand interactions (DeLano, 2002; Pettersen et al., 2004). According to the literature, to identify the accuracy of predicting a ligand binding pose, the RMSD value should be smaller than 2 Å (Kramer, Rarey & Lengauer, 1999). According to the docking results, the most stable structure of NAC in the active region of the protein had a binding affinity value (Δ*G*) of −5.0 kcal mol^−1^, as shown in Table 4. The hydrogen bond could play an important role in ligand binding with protein, as seen in Figs. 8 and 9. The NAC peptide was attached to the receptor protein primarily by the residues ASP-16, VAL-17, and LYS-18 in an active site within the protein. Protein–ligand interactions showed that there are four H-bonds with ASP-16. The first and second are between the H atom in the COOH group and the O atoms in the side chain of ASP-16 (2.6 and 3.3 Å), while the third and fourth are between the H atom in the NH groups and the O atom in the carboxyl group of ASP-16 (2.2 and 3.5 Å), as shown in Fig. 10. In addition, other non-covalent interactions were also observed with VAL-17 (3.5 Å) and LYS-18 (2.8 and 3.0 Å), as shown in Fig. 10. In the docking results for the NAC molecule, the binding energy that corresponded to the lowest RMSD value was −5.0 kcal mol^−1^.

**Table 4:** Binding affinity of different poses of title compound as predicted by AutoDock Vina.

Mode	Affinity (kcal mol^−1^)	Dist. from best mode	
		Rmsd I.b.	Rmsd u.b.
1	−5.0	0.000	0.000
2	−4.8	1.659	1.889
3	−4.5	2.603	4.009
4	−4.4	2.551	4.791
5	−4.3	2.466	5.222
6	−4.3	2.278	5.583
7	−4.2	2.536	3.787
8	−4.1	2.456	7.463
9	−4.1	2.983	4.977

### FT-IR spectroscopy results

**Figure 11:**
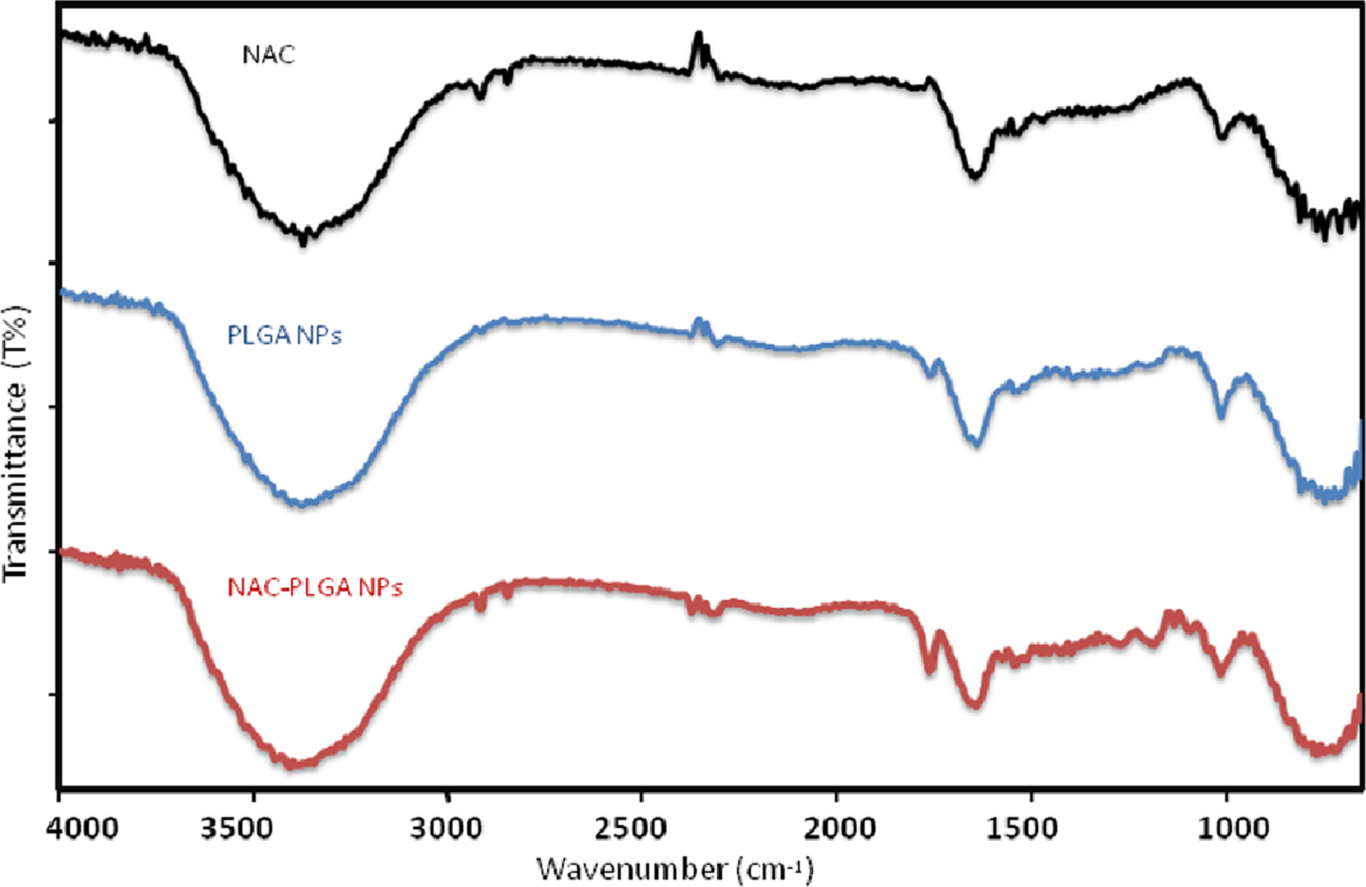
ATR spectra of NAC, NAC-PLGA-NPs, and PLGA-NPs.

The ATR spectra were presented for NAC, PLGA-NPs, and peptide-loaded PLGA-NPs in order to assign characteristic peaks that confirmed the consistency between peptide-loaded PLGA-NPs and PLGA-NPs. The ATR spectra were recorded from 4,000 to 650 cm^−1^, as shown in Fig. 11. The prominent peaks were assigned, and they are tabulated in Table 5. The pure NAC molecule showed the main peaks contributed by the functional groups of molecules, such as carbonyl –C=O stretching (1,649 cm^−1^), –CH, –CH2, and –CH3 stretching (2,920–3,850 cm^−1^), –O–H and –N–H stretching (3,564–3,375–3,343 cm^−1^), and –C–N stretching (1,000–1,339 cm^−1^). The pure PLGA sample (Fig. 11) showed peaks such as –CH, –CH2, and –CH3 stretching (2,700–3,000 cm^−1^), carbonyl –C=O stretching (1,550–1,900 cm^−1^), C–O stretching (1,150–1,300 cm^−1^), and –OH stretching (3,200–3,500 cm^−1^), which were broad. As seen in Fig. 11, the spectrum of NAC-PLGA-NPs was formed with superposition of the characteristic peaks of pure NAC and PLGA components in the ATR spectrum. Because of the low drug concentration in the NPs, the peaks in the region of low wavelength cannot be observed (Erbetta et al., 2012; Gümüşderelioğlu & Deniz, 1999). The absence of some peaks of the drug molecule especially at 1,000–1,339 cm^−1^ is due to the truth that uploading the drug molecule in the PLGA nanoparticle causes shielding of the functional groups of the drug molecule. This fact could be used as an evidence of success in drug loading.

**Table 5:** ATR spectrum of NAC, PLGA-NPs, and NAC-PLGA-NPs.

Erbetta et al. (2012)	Gümüşderelioğlu & Deniz (1999)			This Study			
		dl-lactide	Glycolide	PLA	PLGA NPs	NAC-PLGA-NPs	NAC
3,000–2,700	CH, CH_3_, CH_2_ (stretching)	2,850			2,918	2,916; 2,847	2,920; 2,850
1,900–1,550	C=O (stretch)	1,760	1,750	1,750	1,761; 1,645; 1,537	1,762; 1,643; 1,543	1,692; 1,649; 1,535
1,500–1,250	CH_3_, CH_2_ (symetric angular deformation)		1,540; 1,430	1,450; 1,360	1,395	1,423; 1,395	1,471; 1,397
1,350–1,150	CH_2_, CH (asymetric angular deformation) (wagging)				1,317	1,270	1,252
1,300–1,150	C–O (ester stretch)	1,275; 1,100	1,265; 1,050	1,130; 1,090	1,259; 1,017	1,270; 1,017	1,252; 1,018
750–940	C–H (bending)	940		750	750	767	750

### Determination of encapsulation and loading efficiency of NAC

**Figure 12:**
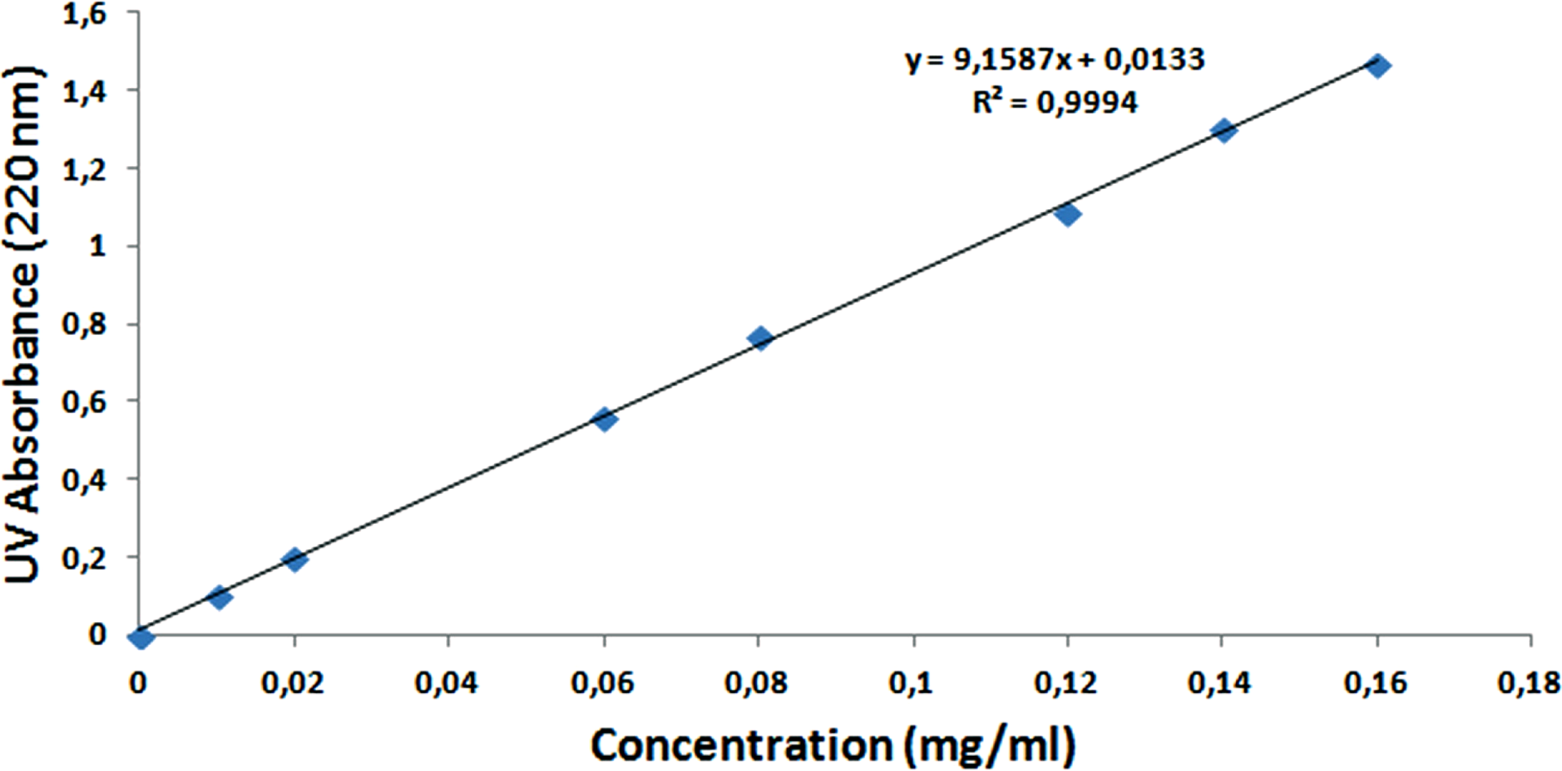
Standard calibration curve of NAC at 220 nm.

To calculate the encapsulation and loading efficiency of NAC, the standard calibration curve of the NAC molecule was formed with different concentrations, as seen in Fig. 12. After the supernatants of NPs were analyzed by UV–Vis spectrometry at 220 nm, the NAC concentration in the supernatant was determined, and the encapsulation efficiency was calculated as 90% by the formulas shown above. The loading efficiency of NAC was calculated as 21%. This means that each 1 mg NAC-PLGA-NP contained 0.21 mg NAC.

### DLS and zeta potential results

The particle size, PdI, size distribution, and zeta potential measurements of the blank PLGA and NAC-PLGA-NPs were conducted using a Zetasizer. The results are shown in Table 6.

**Table 6:** Zetasizer results of nanoparticles.

Nanoparticle	Particle size (nm)	Polydispersity ındex	Zeta potential (mV)
Blank PLGA	222.2	0.030	−8.35
NAC-loaded PLGA	226.9	0.004	−7.41

**Figure 13:**
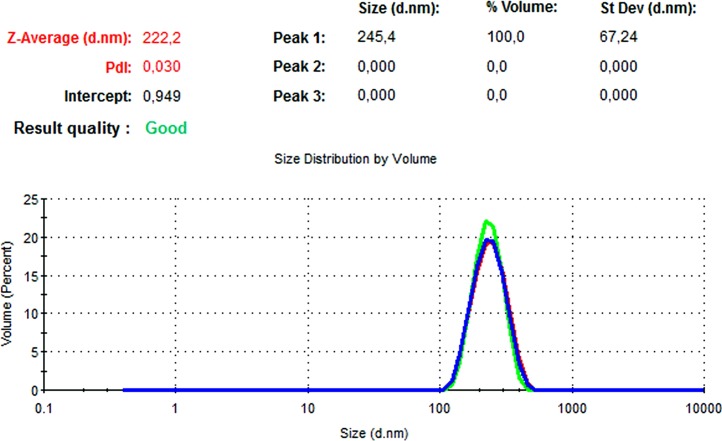
Size distribution graph of blank PLGA-NPs.

**Figure 14:**
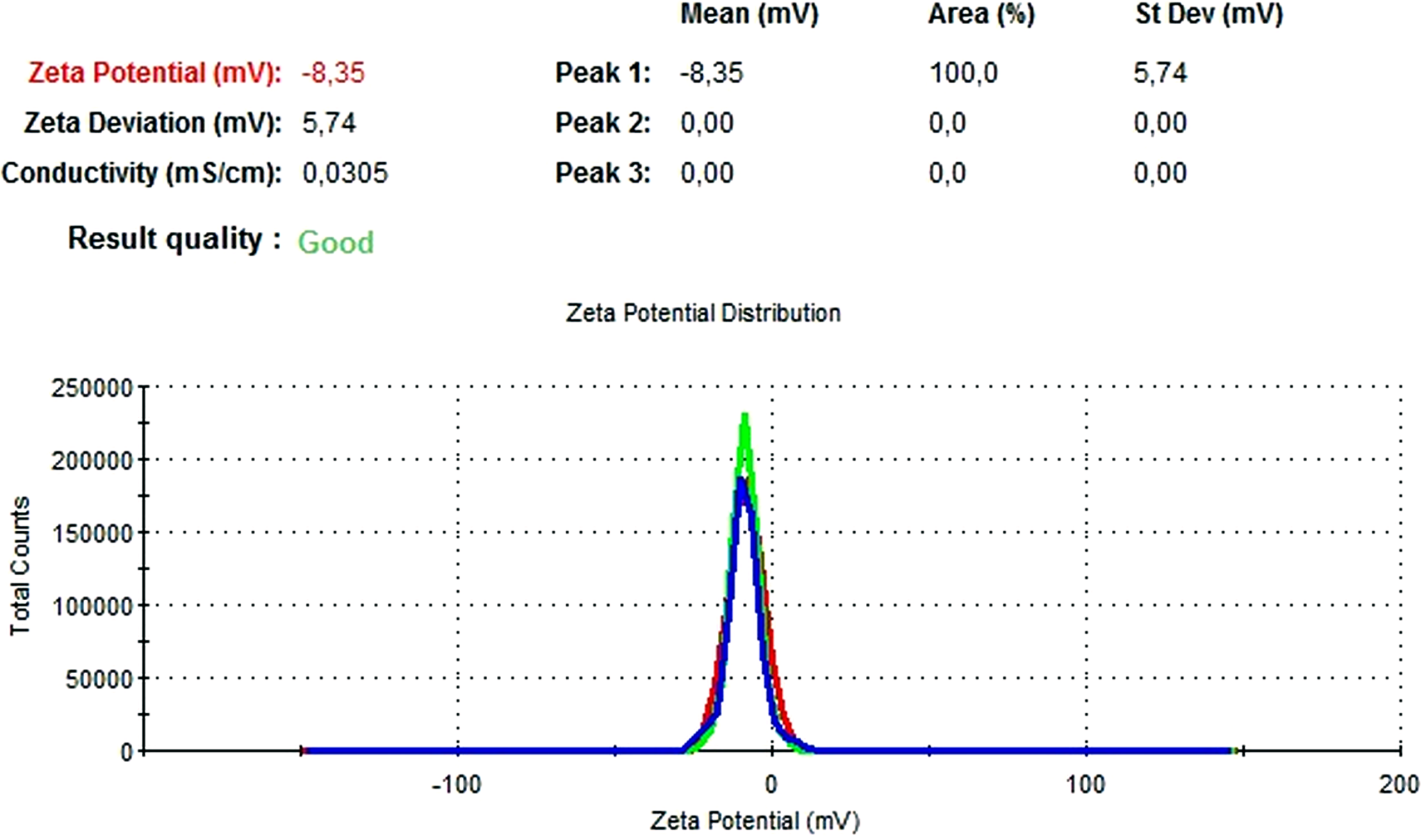
Zeta potential graph of blank PLGA-NPs.

**Figure 15:**
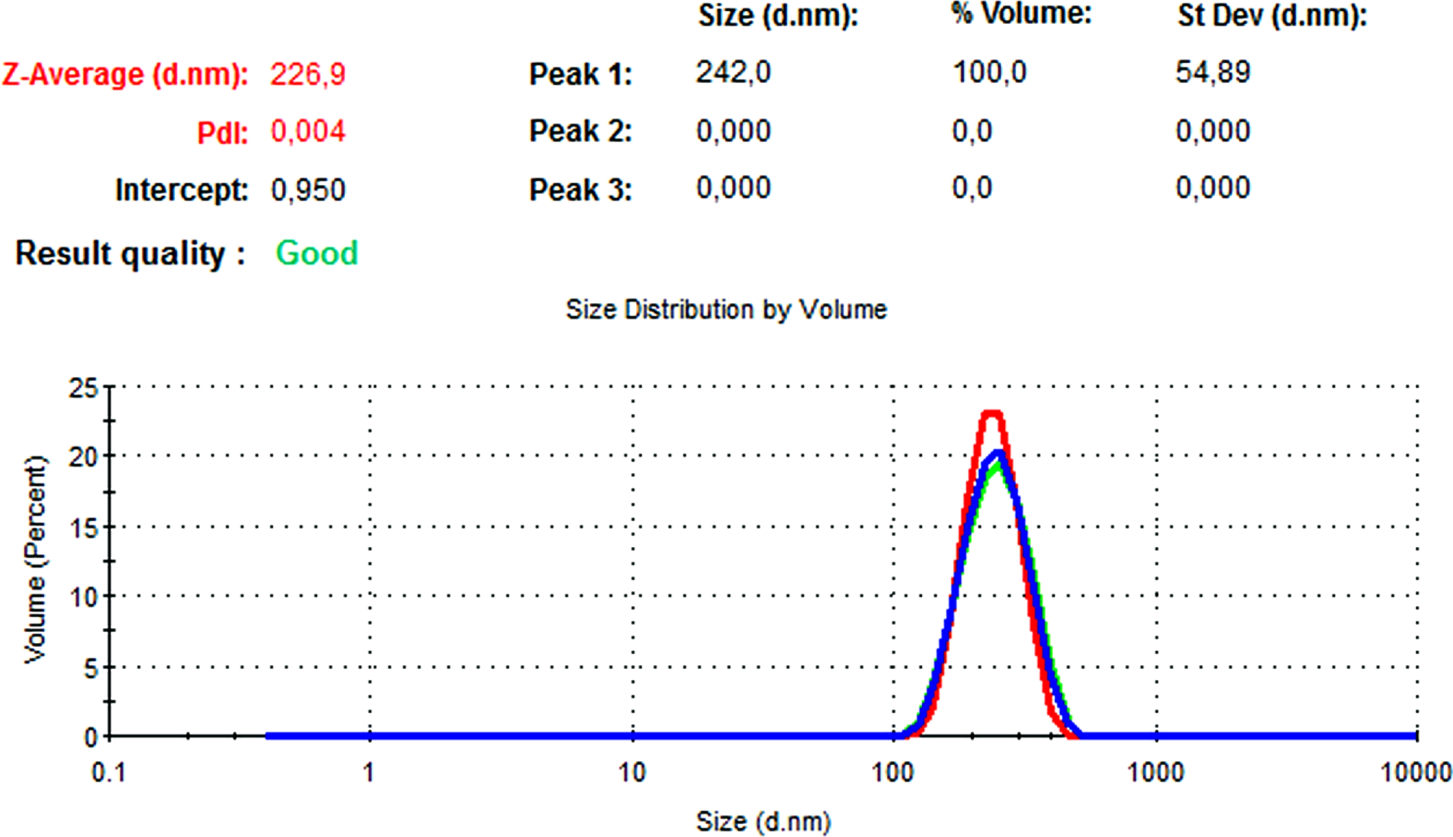
Size distribution graph of NAC-PLGA-NPs.

**Figure 16:**
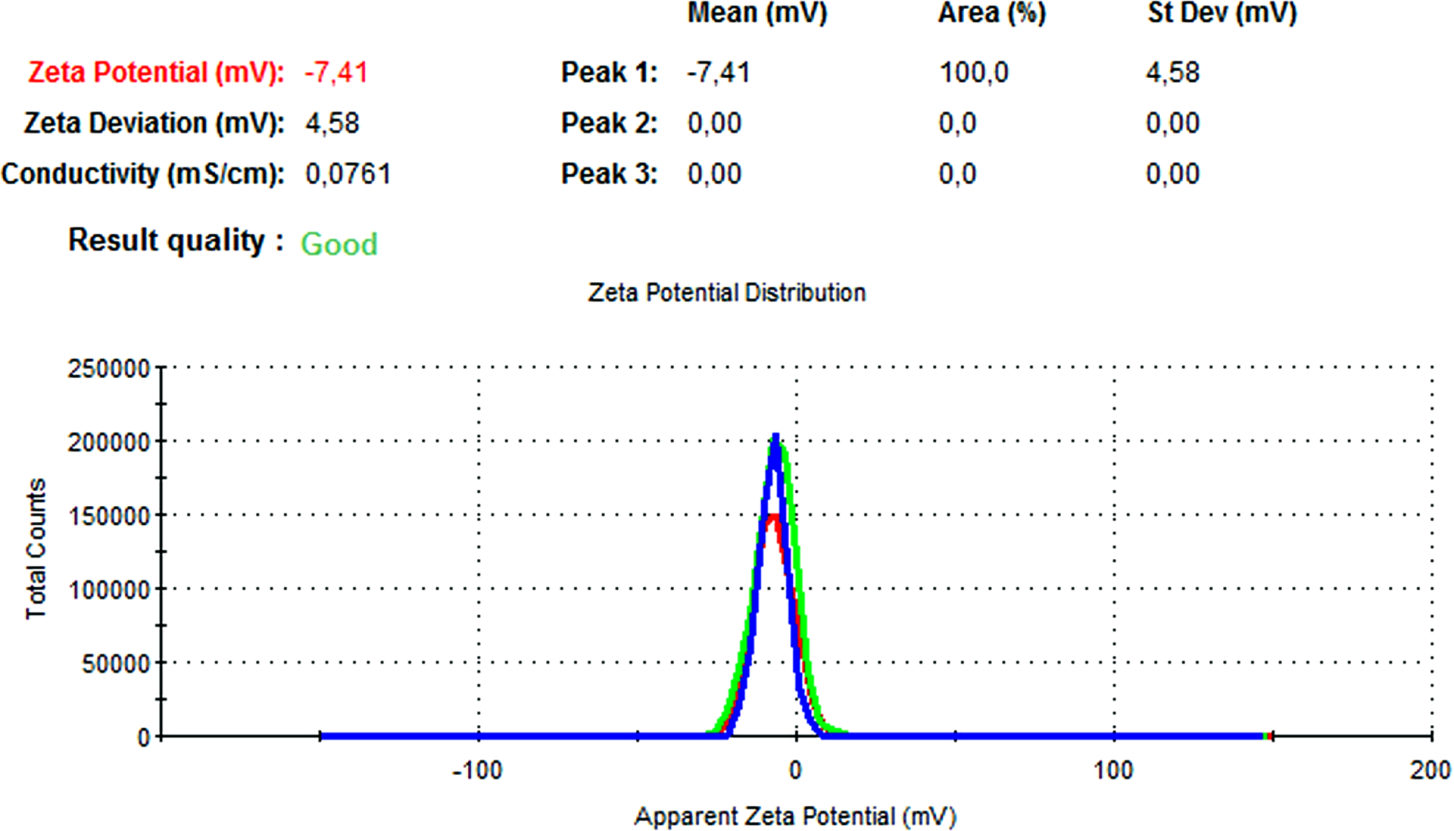
Zeta potential graph of NAC-PLGA-NPs.

In Figs. 13 and 14, the particle size and zeta potential measurements of blank PLGA-NPs are given, respectively. As seen from Fig. 13, blank PLGA-NPs had a narrow size distribution (by volume) of 97.8%, with 0.030 PdI and a 222.2 nm average particle size. In Fig. 14, the zeta potential measurements of blank PLGA-NPs obtained at −8.35 mV are given. The particle size and zeta potential measurements of NAC-PLGA-NPs are given in Figs. 15 and 16, respectively. NAC-PLGA-NPs also had a narrow size distribution (by volume) of 97.7%, with 0.004 PdI and a 226.9 nm average particle size. As can be seen in Fig. 16, the zeta potentials of NAC-PLGA-NPs were obtained at −7.41 mV.

### Results of TEM and energy dispersive X-ray spectroscopy analysis

**Figure 17:**
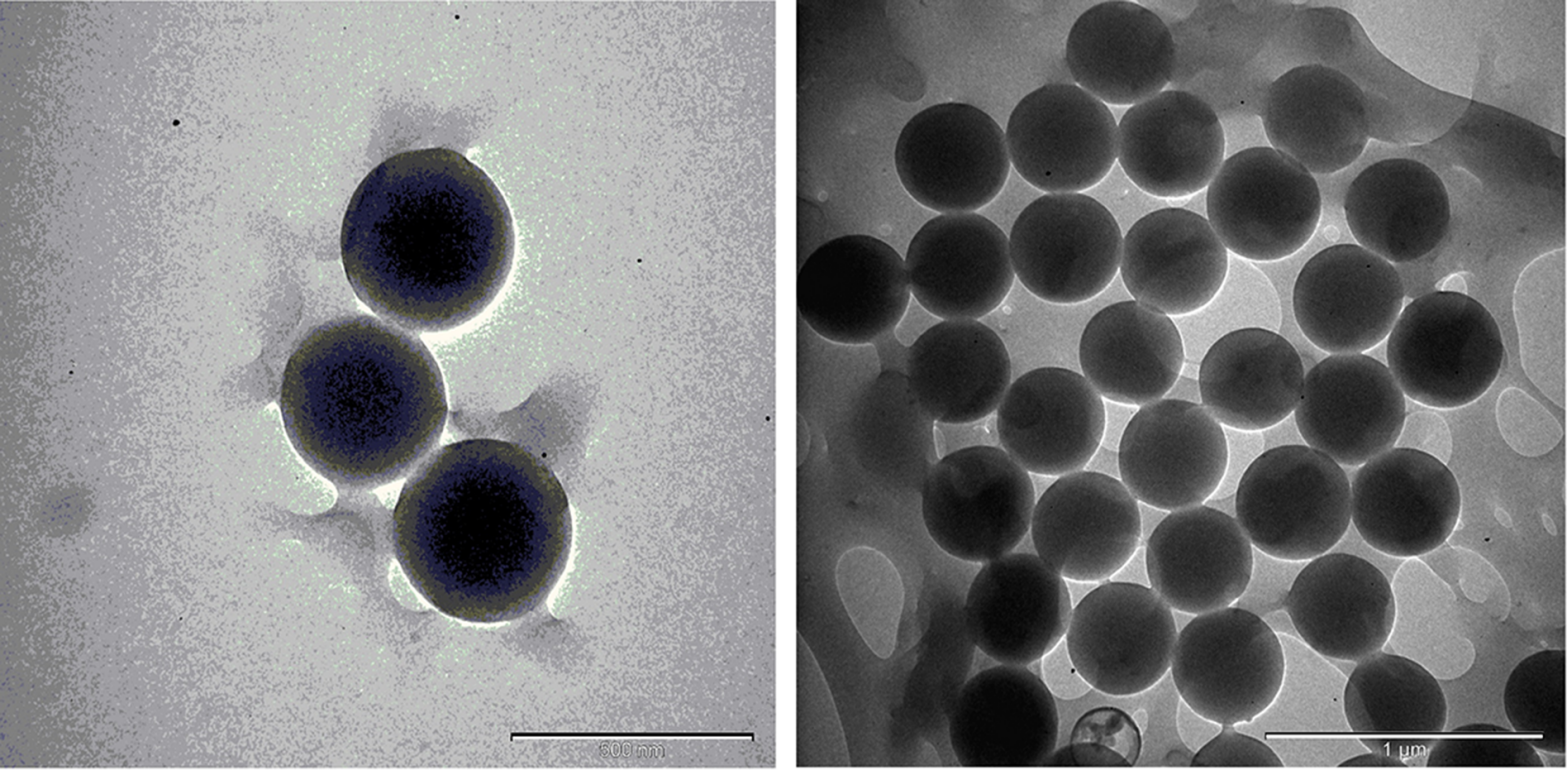
TEM images of NAC-PLGA-NPs.

**Figure 18:**
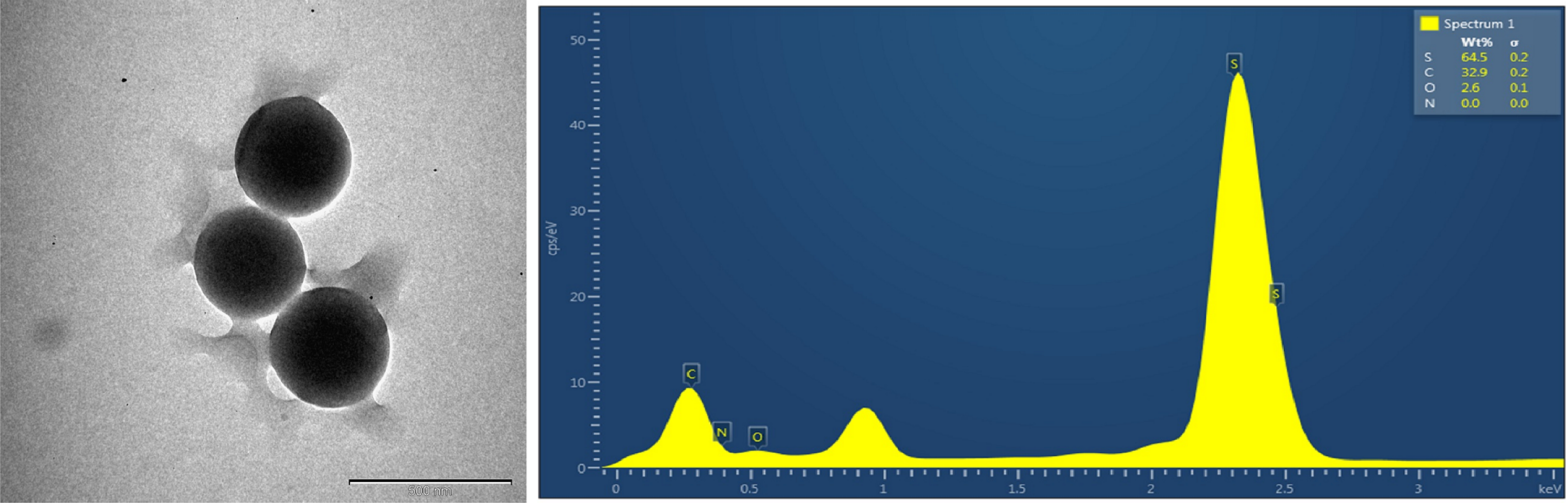
EDS analysis of NAC-PLGA-NPs.

The TEM images are shown in Figs. 17 and 18; the spherical and non-aggregated morphologies of the NAC-PLGA-NPs are clearly displayed from different perspectives. The chemical composition of the NAC-PLGA-NPs was determined via EDS, and the carbon and oxygen peaks are shown in Fig. 18. In addition, a sharp sulfur peak resulted from the cysteine residue of the NAC molecule.

### Cytotoxicity results

**Figure 19:**
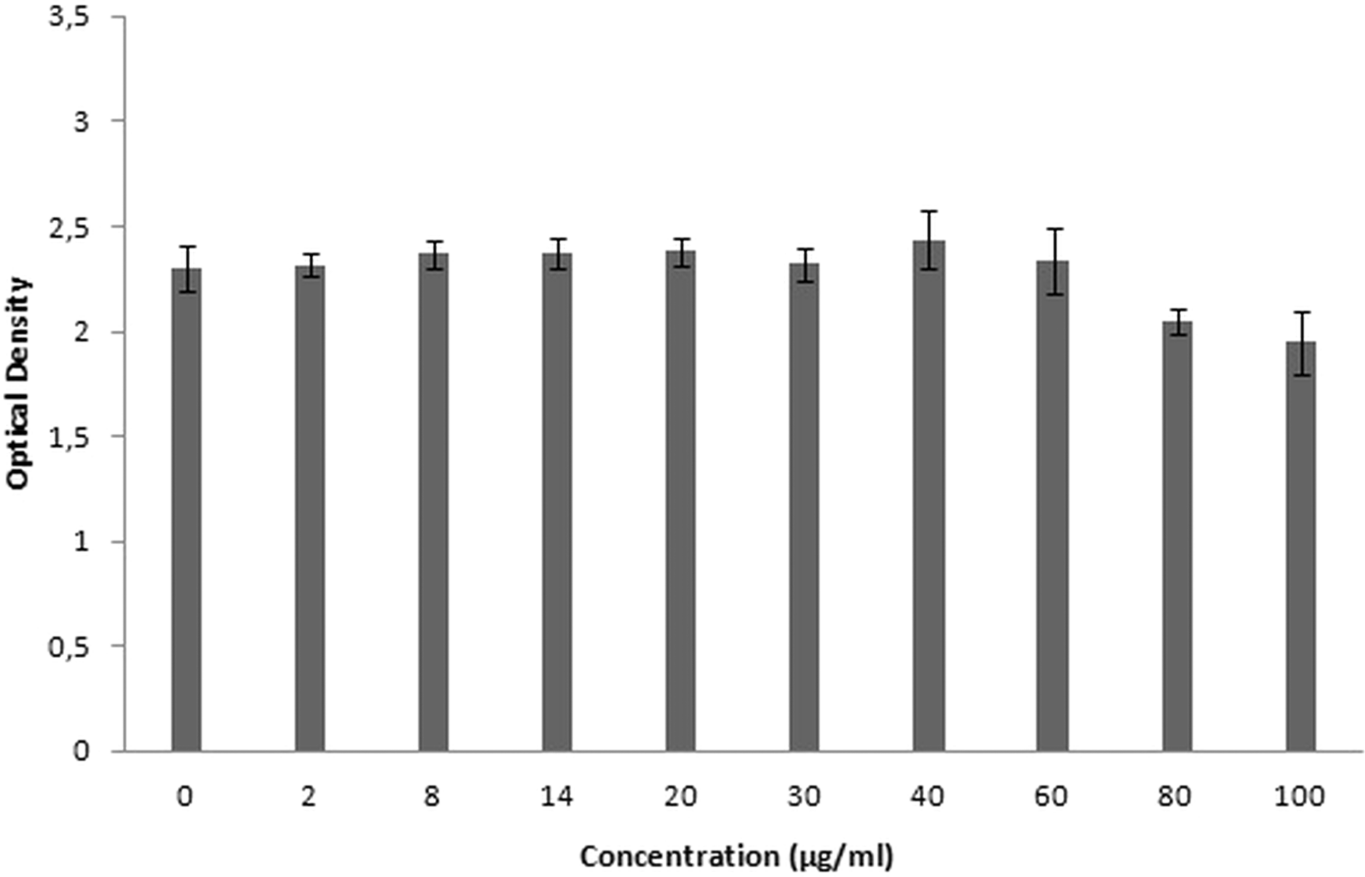
Toxicity of NAC molecule on L929 cells.

**Figure 20:**
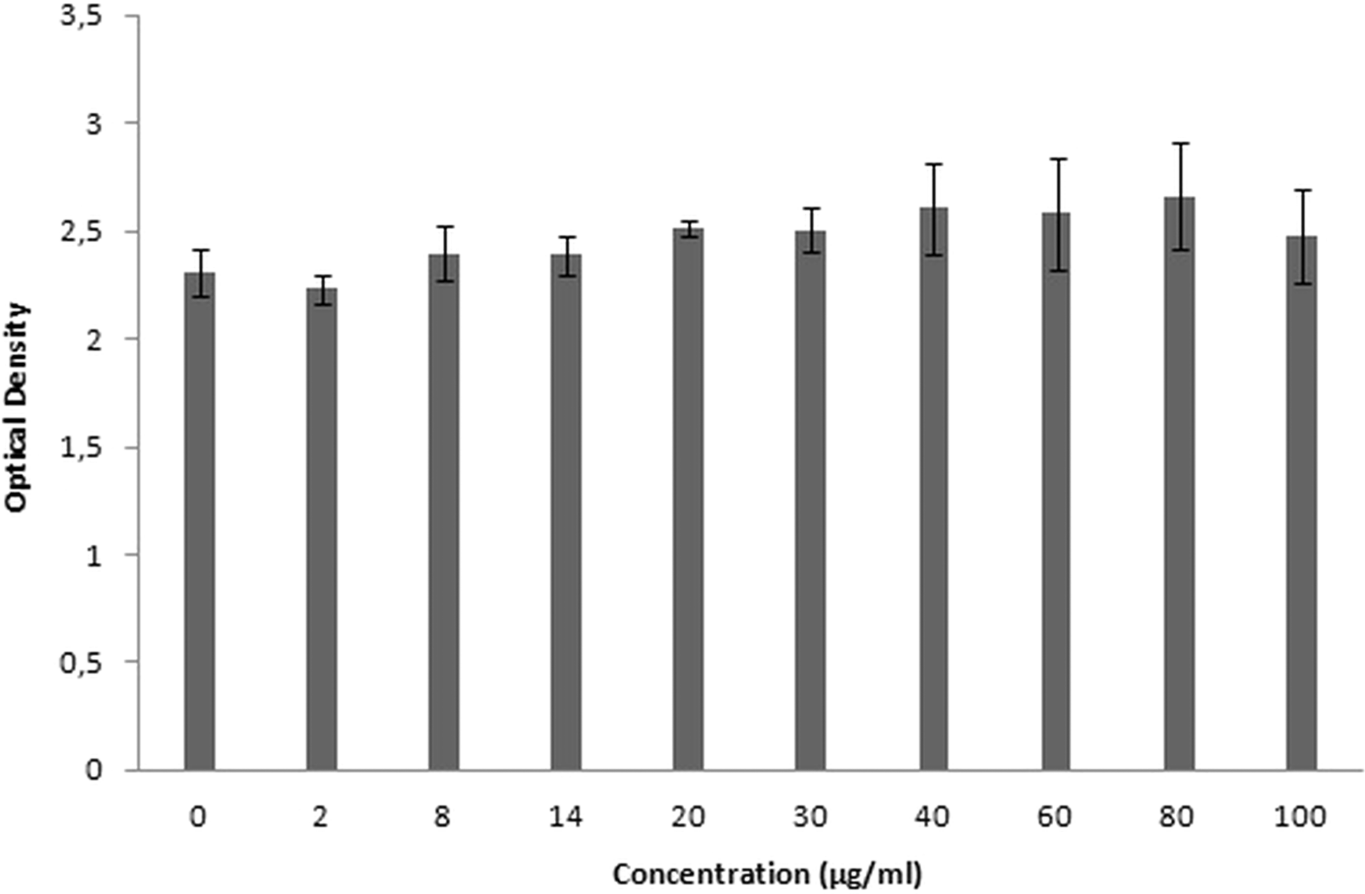
Toxicity of NAC-PLGA-NPs on L929 cells.

XTT is used for cytotoxicity experiments, and it is based on the reduction of reactant to a colorful product in the presence of mitochondrial enzymes of viable cells. Cell viability is proportional to optical density. Figure 19 shows the toxicity of NAC molecules on the L929 cells. As seen in the figure, there is no toxicity of NAC molecules at lower concentrations. Optical density was slightly reduced at higher concentrations. As shown in Fig. 20, NAC-PLGA-NPs did not decrease optical density in the examined concentrations. However, NAC had slight toxicity on cells. When NAC was encapsulated with PLGA-NPs, the toxicity of NAC was reduced. As shown in the figure, there was no toxic effect of NAC-PLGA-NPs on cells in the examined concentrations.

In Table 7, percentage viability values of cells exposed to different concentrations of NAC or NAC-PLGA-NPs are given. As seen in Table 7, only 80 and 100 μg ml^−1^ of NAC reduced cell viability, but according to the literature, more than 80% cell viability is regarded as non-toxic (López-García et al., 2014).

**Table 7:** Viability of cells exposed to different concentrations of NAC or NAC-PLGA-NPs.

Concentration (μg ml^−1^)	NAC	NAC-loaded PLGA NPs
**2**	100.8 ± 2.4	97 ± 3.02
**8**	102.9 ± 2.7	104.2 ± 5.3
**14**	103.1 ± 3.3	103.8 ± 4.1
**20**	103.6 ± 2.9	109.2 ± 1.7
**30**	101 ± 3.4	108.9 ± 4.5
**40**	106 ± 5.9	113.4 ± 9.3
**60**	101.7 ± 6.8	112.2 ± 11.3
**80**	89 ± 2.4	115.8 ± 10.9
**100**	84.7 ± 6.7	107.9 ± 9.4

Therefore, there was no toxicity of the examined concentrations in NAC or NAC-PLGA-NPs.

## Conclusion

In this study, the NAC molecule, which has been investigated as a drug molecule due to its antioxidant and oxidative stress-reducing properties, especially in cataract treatment, was encapsulated with a PLGA polymer in order to increase drug bioavailability.

NAC and NAC-PLGA-NPs were examined for toxicity in L929 cells using FDA-approved PLGA-based NPs as a control, and it was observed that the peptide did not exhibit toxicity when encapsulated with PLGA, even at doses at which the peptide alone reduced cell viability slightly. Encapsulation of the NAC molecule with PLGA has been found to increase bioavailability and biocompatibility. This study may contribute to the design of drugs for cataract treatment with better reactivity and stability.

## Supplemental Information

10.7717/peerj.4270/supp-1 Supplemental Information 1 Potential energy of the system.

10.7717/peerj.4270/supp-2 Supplemental Information 2 System density as a function of time during NPT equilibration.

10.7717/peerj.4270/supp-3 Supplemental Information 3 The protein (excluding hydrogens) RMSD of the N-acetylcarnosine (NAC) molecule.

10.7717/peerj.4270/supp-4 Supplemental Information 4 The standard calibration curve of NAC at 220 nm.

10.7717/peerj.4270/supp-5 Supplemental Information 5 Toxicity of NAC and NAC loaded PLGA nanoparticles on the L929 cells.

## Additional Information and Declarations

### Data Availability

The following information was supplied regarding data availability: The raw data has been supplied as Supplemental Datasets Files.
