# Supplementary figures and images for: Novel NAC-loaded poly(lactide-co-glycolide acid) nanoparticles for cataract treatment: preparation, characterization, evaluation of structure, cytotoxicity, and molecular docking studies
Source: PeerJ. 2018 Jan 30;6:e4270. doi: 10.7717/peerj.4270 (PMC5796282; doi:10.7717/peerj.4270)

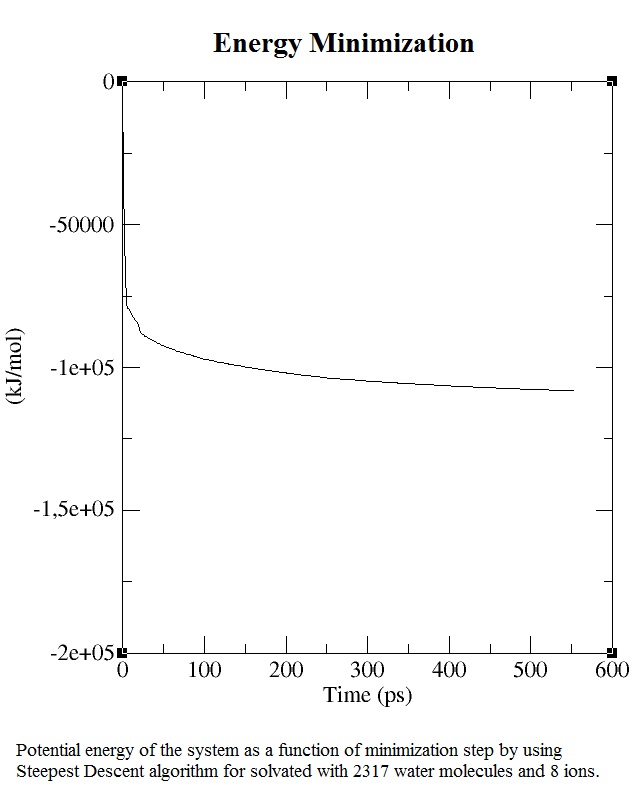

Supplement: Supplemental Information 1 [file peerj-06-4270-s001.jpg]

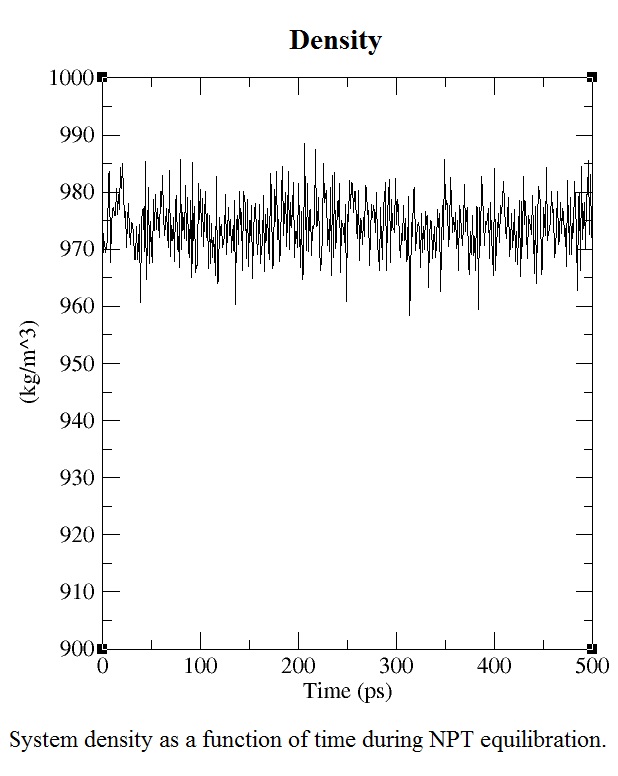

Supplement: Supplemental Information 2 [file peerj-06-4270-s002.jpg]

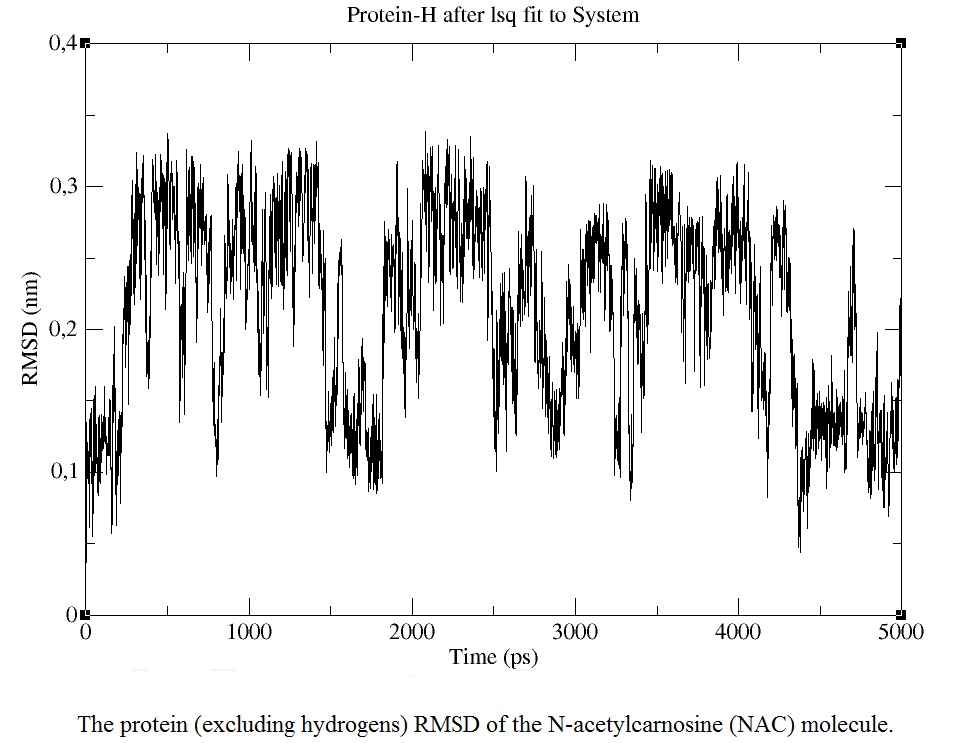

Supplement: Supplemental Information 3 [file peerj-06-4270-s003.jpg]
